# Alginate/k-Carrageenan Interpenetrated Biopolymeric Aerogels for Nutraceutical Drug Delivery

**DOI:** 10.3390/gels11060393

**Published:** 2025-05-27

**Authors:** Alessandra Zanotti, Lucia Baldino, Ernesto Reverchon, Stefano Cardea

**Affiliations:** Department of Industrial Engineering, University of Salerno, Via Giovanni Paolo II, 132, 84084 Fisciano, Italy; azanotti@unisa.it (A.Z.); lbaldino@unisa.it (L.B.); ereverchon@unisa.it (E.R.)

**Keywords:** aerogel, nanostructures, supercritical drying, water uptake, nutraceutical, drug delivery, naringin

## Abstract

Bioactive compounds of natural origin are central to the development of nutraceutical formulations. To improve their stability and to target their delivery to the intestinal or colonic tract, alginate/k-carrageenan spherical gels have been produced at different volumetric ratios (100/0, 70/30, 50/50, 30/70, and 0/100 *v*/*v*), by means of solution dripping and external gelation. Different drying methods were compared, and only through supercritical technologies was it possible to achieve interpenetrated networks that feature nanometric pore size distribution. Hybrid aerogels inherited the most relevant characteristics of alginate and k-carrageenan: they showed remarkable water uptake capacity (e.g., 50.60 g/g), and stability in aqueous media over large timespans. Naringin release tests in simulated intestinal and colonic fluids proved that it is possible to target drug delivery by choosing intermediate alginate/k-carrageenan ratios. Overall, by means of supercritical gel drying, it is possible to generate advanced biopolymeric aerogels, yielding fully natural interpenetrated networks that valorize the most compelling properties of each species involved.

## 1. Introduction

Nutraceuticals, intended as formulations of nutrients that help prevent or treat diseases or illnesses, are attracting interest in the context of Complementary and Alternative Medicine due to being “natural” alternatives to conventional pharmaceuticals [1]. The synergy between nutritional and medicinal benefits, typical of nutraceutical ingredients, results in a broad range of clinically proven positive effects on human health. To name a few, they are administered for mitigating cardiovascular and skin diseases, sleeping disorders, diabetes, and for cancer prevention, etc. [1,2,3,4,5,6]. Among the wide plethora of plant-derived phytochemicals, naringin (4,5,7-trihydroxy flavanone-7-rhamnoglucoside)—extracted from citrus fruit peels—is a flavonoid recognized for its cardioprotective, anti-hyperglycemic, anti-inflammatory, neuroprotective, and antitumoral properties [7,8]. Clinical trials have proved that naringin contributes to reducing ischemic injuries in the intestinal tract [9], decreases the level of proteins involved in intestinal tissue inflammation [10], and limits damage caused by ulcerative colitis [11]. Apart from the nutraceutical industry, naringin has also found a range of applications in the cosmetic and food industries that leverage its anti-oxidant and antibacterial effects [7].

However, just like most flavonoids, naringin is extremely sensitive to light and temperature [12]. To overcome this issue, it is usual to revert to nanoencapsulation methods: namely, nanoemulsions, biopolymeric coprecipitates, dendrimers, liposomes, porous particles, etc., are produced to achieve naringin controlled release without altering its biochemical specifications, yet enhancing its beneficial features and prolonging shelf life [13,14,15,16,17,18]. Biopolymeric porous carriers draw attention in the field of drug delivery thanks to the possibility of bridging ease of production, eco-friendliness, functionality, and pore size distribution [19,20,21,22]. Porous structures are generally produced by means of the sol-gel process, eventually coupled with a drying step—i.e., the removal of the liquid phase from the native gel. The most widespread techniques to achieve gel drying are oven drying, freeze drying, and supercritical gel drying: they, respectively, produce xerogels, cryogels, and aerogels [23]. In general, stress is exerted onto the pores’ walls due to liquid–vapor interfacial forces enhanced during evaporation (in the case of oven drying), or due to liquid–solid adhesive forces associated with ice crystal growth and sublimation during freeze drying [24,25]. In both cases, complete or partial pore collapse is observed, meaning that the original gel hierarchical structure is lost in the process. On the other hand, aerogels are produced by employing supercritical CO_2_ (scCO_2_) to extract the liquid embedded in the native gel. Supercritical fluids have virtually no surface tension, meaning that no stress is applied onto nanopores and gels’ characteristic architecture is not modified during the process [24,26]. Aerogels are suitable candidates for drug delivery purposes, since they offer large pore volume for drug storage; moreover, their hierarchical organization offers the active ingredient a tortuous pathway to overcome, resulting in slower release profiles, or controlled based on pore size distribution and polymer functionality [25,26,27]. Moreover, supercritical gel drying is a feasible tool to produce porous structures for drug delivery purposes on an industrial scale [28]. In this scenario, the choice of raw material is crucial: each polymer organizes in its unique architecture that features characteristic porosity and network interconnection, water uptake capacity, pH- or temperature-responsive properties, biocompatibility, etc. Sodium alginate (Alg) is a water-soluble brown algae-derived polyanion, known for its nontoxicity, biocompatibility, biodegradability, and cellular adhesion; it undergoes ionotropic gelation in the presence of Ca^2+^ divalent cations: ionic bonds are formed, resulting in the typical egg-box configuration [29,30,31]. However, alginate’s structure can accommodate low amounts of water due to its stiffness; this feature can slow down the release of the active compound, limiting its targeting onto specific regions of the gastro-intestinal tract. To address this shortcoming, it is possible to engineer the polymeric carrier with another polymer: the result is an interpenetrated biopolymeric network that brings together the most desirable properties of each species involved. k-carrageenan (k-car) is a linear anionic sulfated exopolysaccharide extracted from red algae, and is highly hydrophilic due to the presence of sulfated groups; it undergoes gelation thanks to K^+^ ions that serve as intramolecular glue for k-car chains [32]. Some attempts to produce Alg/k-car blends are reported in the literature. Mohamadnia and coworkers [33] proved that alginate and k-carrageenan are compatible with each other: gelation processes are non-competing, resulting in a stable interpenetrated network, yet without toxic unreacted crosslinkers. Paşcalău et al. [34] noticed that the k-carrageenan/alginate ratio influences water uptake capacity, as well as blends’ mechanical behavior: the larger this value is, the lower are Young’s modulus and elongation at break. However, k-car could hold water up to 16 times its dry weight; contrarily, calcium-crosslinked alginate only up to 0.5 fold. This trend is confirmed by Yu et al. [35]: alginate provides an “elastic” contribution, whereas k-carrageenan provides the hydrophilic one. The latter is relevant for drug delivery purposes, since it enables tailored drug delivery based on the carrier’s composition.

Considering these results, we aim at producing alginate/k-carrageenan interpenetrated polymeric gels: pure alginate, k-carrageenan, and mixtures of them were obtained. Alginate was chosen due to its gel-forming capability; k-carrageenan, instead, for its capacity to host water. The combination of these two biopolymers could result in an elastic, yet hydrophilic and swellable structure, intriguing for drug delivery purposes. A comparison between different drying techniques was carried out, to prove that only by means of supercritical drying is it possible to appreciate morphological differences between the chosen raw materials. To the best of our knowledge, there are no in-depth studies on the effect of supercritical drying on alginate/k-carrageenan hybrid systems. Eventually, aerogels will be characterized in terms of FESEM, FT-IR, and water uptake capacity, and they were thus loaded with naringin to explore the effect of the gels’ composition on release profiles, with a view to nutraceuticals release to the intestinal/colonic tract.

## 2. Results and Discussion

### 2.1. Comparison of Drying Techniques

Different porous structures have been obtained by means of oven drying, freeze drying, and supercritical gel drying. Each drying technique aims at removing the liquid phase from the starting gel; but, in the first two cases, the liquid–vapor meniscus cannot be neglected, and while water is removed, capillary forces are exerted onto the pores’ walls, leading to their disruption and macroscopic collapse. Contrarily, scCO_2_ has virtually no surface tension, and the solvent embedded in the gel is rapidly, yet efficiently extracted while not affecting the gel’s nanostructured architecture. From a macroscopic point of view, pore disruption determines gel shrinkage. Indeed, shrinkage happens when the “void” embedded in the original gel is substituted by the biopolymer: volume reduction is consequently observed. Therefore, shrinkage measurements provide useful insights about the dried structure’s porosity. The hydrogel beads’ initial diameters were 2.48 ± 0.16 mm, 2.72 ± 0.24 mm, 2.78 ± 0.24 mm, 2.87 ± 0.40 mm, and 2.99 ± 0.56 mm, respectively, for the Alg/k-car volumetric ratios 100/0, 70/30, 50/50, 30/70, and 0/100. The results, obtained according to the procedure mentioned in Section 4, are reported in Table 1.

**Figure 1:**
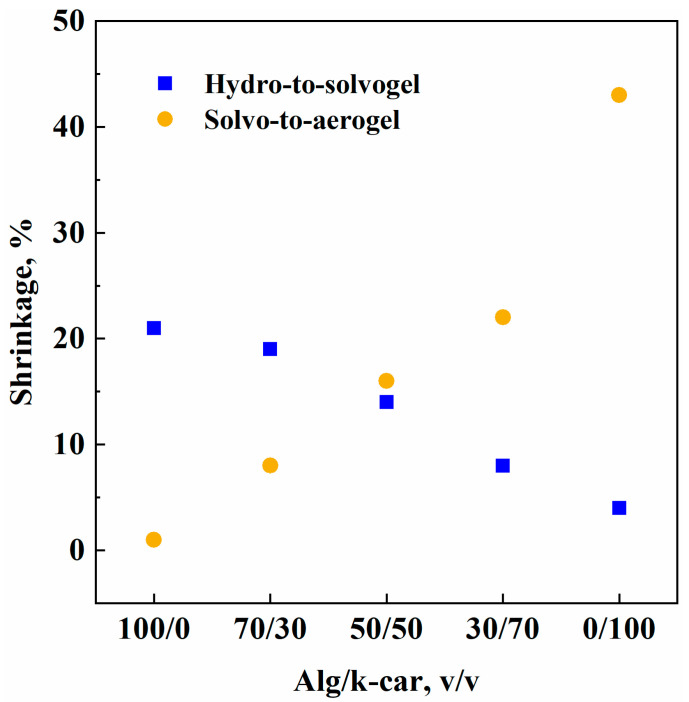
Hydro-to-solvogel and solvo-to-aerogel shrinkage values vs. gel composition.

Some aspects emerge from the data collected in Table 1. In the case of oven drying, the interactions between the two polymers are not strong enough to withstand capillary forces associated with liquid evaporation. Indeed, alginate and k-carrageenan might interact via hydrogen bonding, or to some extent, by association between calcium ions and their respective negative moieties. In both cases, evaporative forces outweigh the gels’ strength. Similarly, the cryogels’ shrinkage values are basically invariant with composition. Indeed, during freeze drying, ice crystals are formed, and they serve as a pores template prior to sublimation: this process pushes the frames of the biopolymeric network, leading to smaller pores’ collapse regardless of the composition. To further validate this hypothesis, the pure carrageenan beads produced, as presented, were basically pulverized during liquid removal: network strength was not stable enough to yield a continuous structure. Therefore, it was not possible to obtain particle size distribution for k-car xerogels and cryogels. Contrarily, supercritical drying is much gentler on the delicate biopolymeric structure: k-car beads were produced successfully. Although the calculated shrinkage value is significant (i.e., 45%), it is consistent with information on the same material in the literature [36]. However, the shrinkage values reported in Table 1 relative to supercritical drying hide two different contributions that are factored in: the first associated with hydro-to-solvogel transition (bound to solvent exchange); the second with solvo-to-aerogel transition, related to the actual gel drying. To have an in-depth acknowledgement of these phenomena, the diameter shrinkage values were obtained step by step. The results are reported in Figure 1.

Evidently, alginate and k-carrageenan suffer shrinkage in different steps of the process. On the one hand, alginate is much more liable to solvent exchange than k-carrageenan: namely, shrinkage values plummet from about 20% to 4%. On the other hand, the response is inverted for the solvo-to-aerogel transition. For k-carrageenan, it is possible that the water–polymer interaction forces are as strong as ethanol–polymer interactions: however, when ethanol is extracted, chain–chain attraction forces are augmented, and this brings about structural compaction, thus shrinkage. Alginate, instead, is more sensitive to solvent exchange: a 20% shrinkage is observed, probably due to a slight network reconfiguration during liquid substitution. Nevertheless, once the structure is stable, it does not suffer solvent extraction: a negligible shrinkage value of 1% is obtained after supercritical drying. Shrinkage values for both steps follow an almost linear trend with k-car concentration: namely, it decreases for solvent exchange, and it increases for supercritical drying. Hybrid gels behave intermediately between alginate and k-carrageenan, and shrinkages’ linearity represents an equal contribution of the two chosen biopolymers to processing steps.

**Figure 2:**
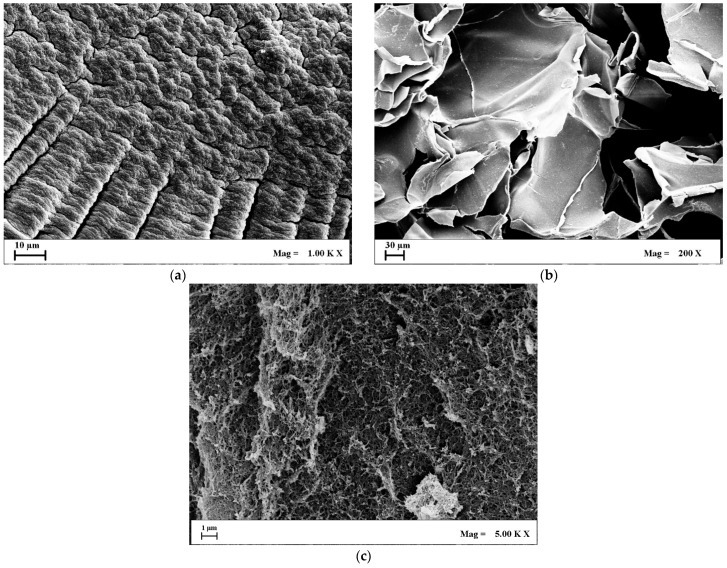
Landscape FESEM images of 50/50 Alg/k-car: (**a**) Xerogels; (**b**) Cryogels; (**c**) Aerogels.

To analyze the effect of the drying method on the configuration of the biopolymeric network, FESEM images were taken. For the sake of clarity, only Alg/k-car 50/50 porous structures are shown in Figure 2, although the proposed considerations are valid also for the other produced samples.

**Figure 3:**
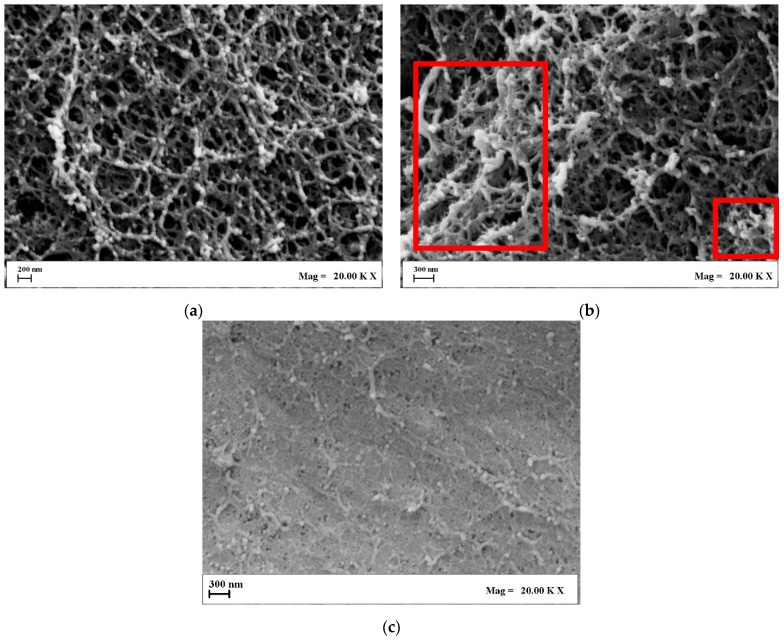
In-detail FESEM images of Alg/k-car aerogels: (**a**) 100/0; (**b**) 50/50; (**c**) 0/100.

Landscape images of the produced porous structures confirm the general trend proposed in the scientific literature. Namely, oven drying yields nonporous devices (Figure 2a), whereas freeze drying generates voids of approximately hundreds of microns (Figure 2b), organized in the usual “crumpled paper” morphology typical of cryogels. On the other hand, aerogels display remarkable “roughness”, and smaller pores are kept intact during supercritical drying (Figure 2c). Morphological analysis is consistent with gel shrinkage considerations. Xerogels have virtually no pores, and every available void present in the native gel is sacrificed for compaction, thus volume reduction. Cryogels, instead, offer macropores that correspond to the ice templates sublimated during the drying step. Indeed, a larger void fraction exists, reflecting smaller shrinkage values. Aerogels show no damage to the original nanostructure, meaning that the liquid embedded in the gel has simply been swapped with air. Consequently, aerogels are the samples that display the lowest shrinkage value. In addition, there is no possibility to differentiate the morphological contribution of alginate from that of k-carrageenan by looking at the FESEM micrographs of xerogels and cryogels. Network compaction led to conglomeration, causing the two biopolymers to lose their attributes. In the case of aerogels, instead, it is possible to evidence the fishnet-like structure of alginate [37], as well as denser zones typical of k-carrageenan [38], meaning that these species are well blended and interconnected, possibly resulting in maintaining—yet, discerning—both the alginate’s elasticity and k-carrageenan hydrophilicity. Indeed, the effectiveness of supercritical drying in preserving the nanoscale interpenetrated network has been proved for other biopolymeric blends [37,39], but not for species that respond so conversely to contact with scCO_2_. To dive deeper into the morphological contributions of each biopolymer to the interpenetrated network, FESEM nanoscale observations of the produced aerogels are shown in Figure 3. For the sake of clarity, alginate, k-carrageenan, and the 50/50 mixture of them are reported, establishing that the relative abundance of the carrageenan’s and alginate’s zones is directly proportional with their ratio in the initial blend.

**Figure 4:**
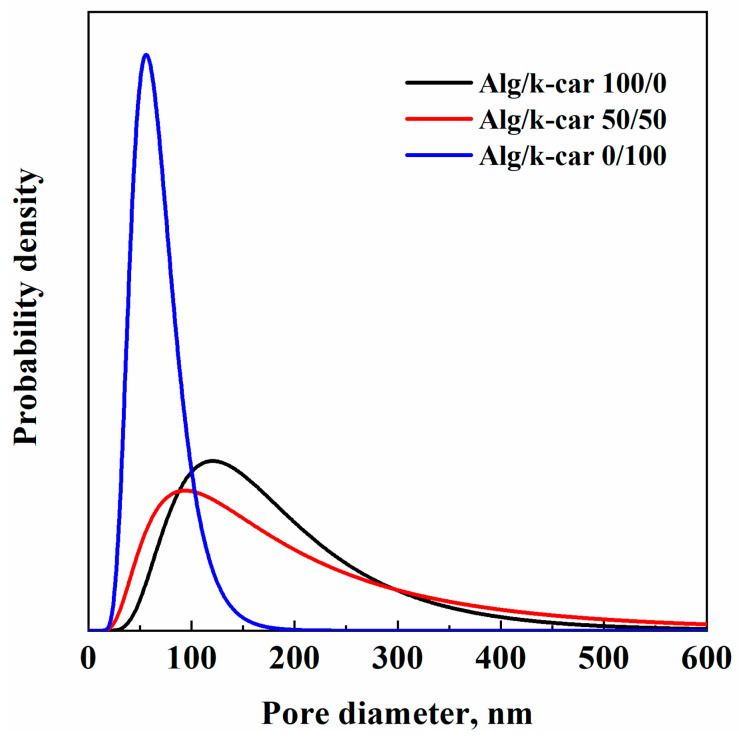
PSD of Alg/k-car aerogels.

As shown in Figure 3, blending alginate and k-carrageenan was successfully carried out, although the two biopolymers organize in completely different morphologies. Indeed, alginate aerogels offer a regular pearl-necklace morphology, in which the polymeric chains are well-interconnected in every direction (Figure 3a). In contrast, k-carrageenan is much denser—coherently with the measured shrinkage values—and exhibits more closed pores (Figure 3c). The cross-section tends to flatten with respect to the alginates, although nanopores are visible and intact, still being viable hosts for water intrusion and drug loading. The Alg/k-Car 50/50 blend exposes both the fishnet-like architecture of alginate and some densified regions typical of k-carrageenan (evidenced by the red frames in Figure 3b). Morphologically speaking, it is evident that the interpenetration of the chosen polymers took place successfully, and the typical features of both were preserved thanks to supercritical gel drying. It is possible to combine biopolymers that undergo different gelation dynamics and respond differently to processing steps. Complementary to FESEM observations, pore size distributions (PSD) were also evaluated. The results of these computations are reported in Figure 4.

PSD is affected by the alginate/k-carrageenan ratio. The pore mean diameter values are about 121 nm, 94 nm, and 56 nm, respectively, with increasing k-carrageenan relative concentration. All the samples show, on average, nanosized pores. However, pure alginate and the 50/50 mixture exhibit pronounced skewness, due to the existence of a supramolecular hierarchical structure. Moreover, network densification due to k-carrageenan addition is evident by mean pore diameter reduction, and the widening of the distribution with respect to pure alginates. This behavior lies in the fact that there are denser zones—featuring, on average, smaller inner pores—resulting in other polymer-lean regions. Potentially, this shift of mean pore size and curve widening could result in an initial faster water intrusion—due to the “polymer-lean” regions, but also in a pronunciation of water uptake capacity due to k-carrageenan smaller pores. On the other hand, for pure k-carrageenan aerogels, there are fewer, yet smaller pores due to the absence of a structured network like alginate’s; but distribution is narrower. Overall, PSD evaluation is useful for drug delivery purposes: by engineering the pores’ width, and thus aerogel composition, release can be tailored.

Indeed, the preservation of the nanoporous network is a desirable feature for drug delivery applications: a larger drug storage volume is available, and release can be controlled based on either pore size distribution or gel composition [25]. Overall, supercritical drying guarantees control over morphological features, as well as pore size distribution. Therefore, further analysis will be carried out only on aerogels, and for the sake of clarity, only alginate, k-carrageenan, and their 50/50 *v*/*v* blend will be considered.

### 2.2. Water Uptake

**Figure 5:**
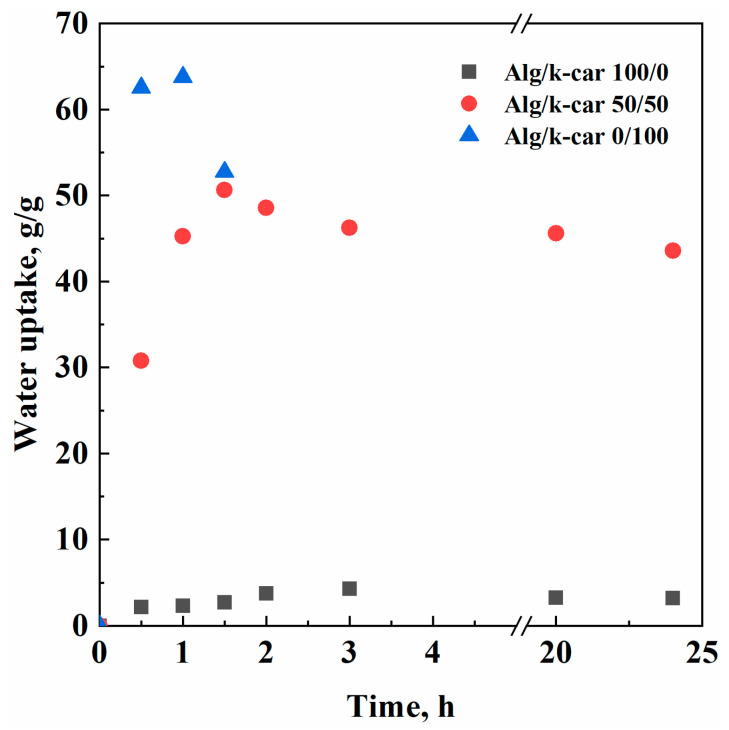
Water uptake experimental points.

It should be foreseen that drug release can be dependent on aerogels’ capacity to take and hold up water. Water uptake kinetics are reported in Figure 5, and the most significant findings are summed up in Table 2.

Aerogels’ water uptake capacity can be considered as the result of different counteracting mechanisms. The network swells due to absorption, up to the moment in which polymer–water interactions or the mechanical stress exerted by the fluid’s surface tension outweigh chains’ elasticity. In that moment, aerogels are eroded, losing part of their initial weight. Otherwise, if the structure is not disrupted, a plateau value can be expected. As shown in Figure 5, in the case of alginate aerogels, there is a limited mass loss, probably due to unreacted chains that are redissolved in water during imbibition. However, the interconnected network is more stable than elastic, resulting in a system that hosts small amounts of water (i.e., a maximum of 4.3 g/g), but is resistant over time. On the other hand, k-carrageenan is surprisingly hydrophilic: it can absorb water up to 63.77 g/g. Nevertheless, the combination of carrageenan aerogels’ small pore size and their high hydrophilicity results in their dissolution in short times: a significant mass loss was measured after 1 h. The Alg/k-car 50/50 mixture, instead, is as stable as alginate, but its water absorption capacity takes after the carrageenan fraction. In terms of water uptake and resilience, the hybrid produced outperforms some other k-carrageenan aerogel beads proposed in the scientific literature, whose maximum water uptake capacity is about half of the one obtained in this work [40]. Overall, it is possible to combine the most useful features of k-carrageenan and alginate to engineer a porous device intended for drug delivery applications. Having a full prospect of aerogel composition, pore size, and water uptake capacity, it is possible to tailor the rate of drug release in the intestinal tract.

### 2.3. Naringin Loading

Once the blank aerogels were prepared and their properties assessed, a set of naringin-loaded aerogels was obtained following the procedure described in Section 4. Prior to further analysis, loaded aerogels were weighed and compared with blank samples: a mass increase of about 10% was observed, meaning that naringin loading took place successfully and that it was not extracted by scCO_2_, in the operating conditions adopted (i.e., 200 bar and 35 °C). In fact, supercritical carbon dioxide acts as an antisolvent for naringin [41].

#### 2.3.1. FT-IR Analysis

**Figure 6:**
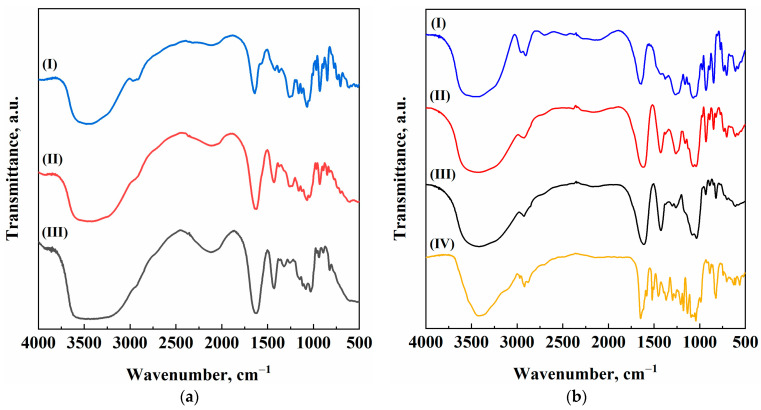
FT-IR spectra of (I) Alg/k-car 0/100, (II) Alg/k-car 50/50, (III) Alg/k-car 100/0, (IV) Naringin: (**a**) Blank aerogels; (**b**) Loaded aerogels.

First, FT-IR spectra were obtained for the produced samples, to check the kind of chemical interactions that take place between the active principle and the polymers’ functional groups. Figure 6 shows and compares the FT-IR spectra of the blank and loaded aerogels.

In Figure 6a, it is possible to evidence the characteristic peaks of both alginate and k-carrageenan. For sodium alginate blank aerogels, the broad band at about 3400 cm^−1^ is associated with stretch vibrations of the hydroxyl groups. The sharp signal at about 1600 cm^−1^ belongs to the asymmetric stretching of –COO^−^ groups, whereas the one located around 1410 cm^−1^ is bound to the symmetric stretching of the same functional group [42]. Similarly, k-carrageenan aerogel shows the same –OH characteristic peak between 3600 and 3200 cm^−1^. Other significant peaks belong to C–H stretching (2930 cm^−1^), asymmetric stretching of sulfated groups (1241 cm^−1^), and glycosidic bonds (1069 cm^−1^) [43]. Consistently, the blended aerogel responds with a hybrid spectrum with the relevant peaks of both alginate and k-carrageenan. The naringin-loaded aerogels, instead, show some slight changes in the FT-IR spectrum (Figure 6b), when compared with their blank counterpart. First, the naringin spectrum features characteristic peaks. Once again, there is a broad band between 3600 and 3200 cm^−1^, due to O–H stretches, although it is sharper than the one shown by the biopolymers due to different molecular configuration. There is a typical peak at 2920 cm^−1^ for C–H stretching vibrations, and others are located at 1645 and 1040 cm^−1^ (carbonyl and –C–O– groups, respectively). The “finger-like” shape of the region between 1400 and 1000 cm^−1^ can be reconducted to the presence of aromatic rings in the naringin molecule. By comparing Figure 6a,b, it is possible to evidence that the loaded aerogels show a “new” peak with respect to the blanks, located at 2920 cm^−1^. Also, slight modifications in the fingerprint region can be appreciated [44]. The presence of naringin characteristic peaks in the FT-IR traces is a reflection of the fact that naringin has not been extracted from the solvogel during supercritical drying, confirming the correctness of the operating conditions selection.

#### 2.3.2. Naringin Release

**Figure 7:**
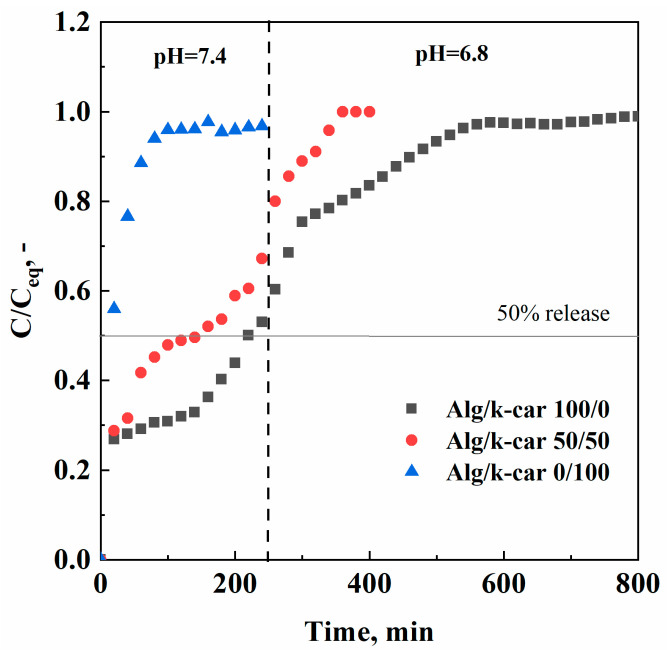
Naringin release profiles at pH = 7.4 and pH = 6.8, for the Alg/k-car 100/0, 50/50, and 0/100 aerogels.

Once the characterizations of interest were carried out, naringin release profiles were obtained. Specifically, the Alg/k-car 100/0, 50/50, and 0/100 aerogels were immersed in PBS at sequentially different pH values (i.e., 7.4 and 6.8), to simulate naringin release in the intestinal and colonic tract. The experimental points obtained are reported in Figure 7.

As shown in Figure 7, active compound release can be tailored depending on if the target is the small or the large intestine. The time needed to release the 50% of the loaded naringin is about 10 min, 2.5 h, and 4 h, respectively, for Alg/k-car 0/100, 50/50, and 100/0 aerogels. For instance, if naringin should be delivered immediately to the small intestine, carrageenan aerogels are indicated for such purpose. Almost all the active principle is released in less than two hours, following a simple exponential trend, with no significant burst release (i.e., an initial linear trait). This result is consistent with the considerations carried out on water uptake measurements: k-carrageenan solubilizes shortly after 1 h, leading the active principle to be released rather than delivered. In this case, the contribution of mass transfer resistances can be almost neglected, since matrix erosion evolves faster than naringin diffusion in the polymeric network. Erosion dominates the overall delivery, due to both k-carrageenan solubility in water, and its structural configuration (i.e., small pore size). On the other hand, naringin delivery from alginate aerogels follows different rates. First, there is a burst release in the first 20 min; then naringin undergoes a slower delivery, since it must overcome the tortuous path offered by the aerogel structure at different rates based on pore size distribution, that, as described before, is quite wide due to the hierarchical architecture. Therefore, different phenomena take place, and the rate of release is thus affected. It can be imagined that water swells up the beads, naringin is desorbed from the solid surface and moves to the embedded water, and then it slowly moves across the aerogel’s porosities, right up to the bulk phase. In this case, it is possible that the contribution of diffusion phenomena outweighs the one related to matrix erosion, as the water uptake measures proved that alginate does not undergo significant mass loss over time [32]. This kind of unbalanced mechanism can be exploited to achieve controlled drug delivery. Once again, the interpenetrated network (i.e., the Alg/k-car 50/50 blend) behaves in-between the pure aerogels. The blend exhibits a release rate similar to that of alginate aerogels, although slightly faster: for instance, the time needed to release 50% of the bioactive compound is 2.5 h and 4 h for the blend and alginate aerogels, respectively. These values are very different from the 10 min needed in the case of k-carrageenan beads. Keeping the focus on the blend, naringin delivery takes place faster than in the case of pure alginate, due to the solubilization of the carrageenan fraction; but, thanks to the crosslinked alginate structure, for instance, it is possible to deliver about 65% of the active compound in the small intestine, and the residual amount in the colonic tract. The employment of porous structures delays the naringin plateau value: for instance, Adami et al. [41] produced naringin breathable powder, whose plateau was reached after only one hour. It is thus confirmed that the utilization of porous structures provides control over the rate of release of the bioactive principle.

## 3. Conclusions

In conclusion, the results proposed in this work prove that supercritical gel drying successfully yields interpenetrated biopolymeric aerogels, which are morphologically regular despite the counteracting behavior of alginate and k-carrageenan. It is possible to obtain hybrid nanoporous structures that leverage alginate’s network elasticity and k-carrageenan’s hydrophilicity and water uptake capacity. The combination of these properties results in a tailored drug release depending on aerogel composition. Naringin delivery can be controlled by changing the ratio between alginate and k-carrageenan, thus deciding how much active principle should be delivered resulting from either diffusion phenomena or release due to erosion processes. Definitively, it is possible to obtain fully natural engineered formulations intended for nutraceuticals delivery. In perspective, a protective layer to guarantee resistance to gastric fluids will be added, advancing the utilization of porous particles for targeted drug delivery.

## 4. Materials and Methods

### 4.1. Materials

Sodium alginate, k-carrageenan, calcium chloride (CaCl_2_), potassium chloride (KCl), and naringin were bought from Sigma Aldrich (St. Louis, MO, USA). Ethanol was purchased by Carlo Erba Reagenti (Chaussée du Vexin, France). Water was produced in a home-made distillation column, and its measured pH was 6.88. CO_2_ was supplied by Morlando Group Srl (Napoli, Italy).

### 4.2. Methods

#### 4.2.1. Gel Preparation

**Figure 8:**
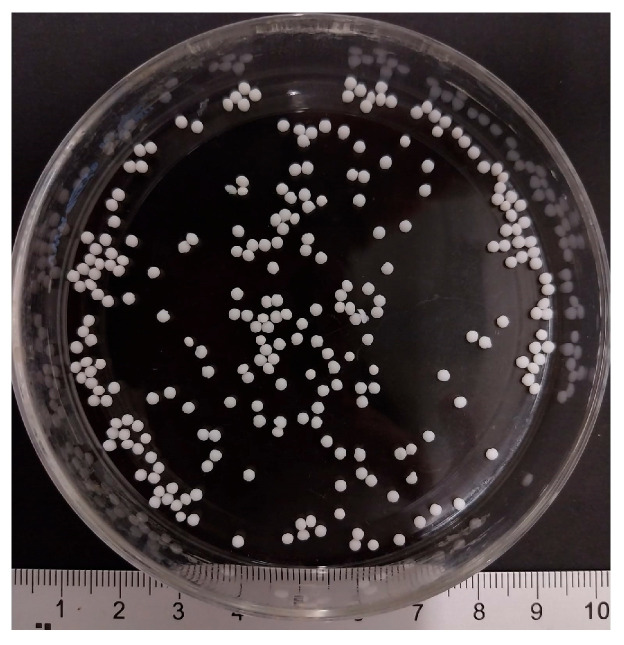
Alg/k-car 50/50 aerogels.

Hydrogel beads were prepared by solution dripping and ionotropic gelation. For both alginate and k-carrageenan solutions, a concentration of 2% *w*/*v* was chosen after preliminary trials. Sodium alginate and k-carrageenan 2% *w*/*v* aqueous solutions were prepared separately at room temperature, gently stirred to avoid air bubbles’ entrapment in the polymeric solution. To obtain the 50/50 Alg/k-car blend, the two solutions were mixed by a 1:1 volumetric ratio. Once the desired solutions were prepared, they were extruded using a syringe pump (KF Technology, mod. AL-1000, Roma, Italy) through a 27G needle, so that beads could be small enough with a view to oral drug delivery. Flow rate was set at 0.25 mL/min, to achieve correct solution dripping without incurring in filament generation (higher flow rates)—also taking into account solutions’ viscosity and resistance to flow. Solution droplets were set to fall into aqueous coagulation baths, whose composition was CaCl_2_ 2 M for alginate spheres, KCl 2 M for k-carrageenan beads, or equimolar mixtures of them for the hybrid solutions. After extrusion, the beads were aged in the coagulation bath for 2 h to ensure complete gelation; then, they were rinsed with distilled water to remove unreacted salts. For xerogels and cryogels production, no further post-processing was applied. Xerogels were obtained by locating hydrogels into an oven, kept at 40 °C and at 0.5 bar overnight. For cryogels production, a cryostat (LyoQuest-55 Plus ECO, Seneco srl, Milan, Italy) was employed, set at −50 °C and under vacuum for 12 h. To obtain aerogels, hydrogels were converted to solvogels by gradual solvent exchange with ethanol. Ethanol/water solutions were employed, at gradually increasing ethanol concentration (i.e., 10, 30, 50, 70, 90, 100% *v*/*v*). Each step was set to last for 1 h, exception made for the pure ethanol exchange that lasted overnight. Then, supercritical drying was performed at 200 bar and 35 °C, for 1.5 h plus a slow depressurization step. This process was carried out in a lab-scale supercritical drying unit, whose details are already reported elsewhere [24]. For clarity purposes, a digital image of Alg/k-car 50/50 aerogels is reported in Figure 8.

For the preparation of naringin-loaded gels, a ratio of 1:10 *w*/*w*_polymer_ was chosen. Naringin was suspended in aqueous environment: then, biopolymers were added. The following steps are the same as the one applied for blank aerogels preparation.

#### 4.2.2. Characterizations

Hydrogel beads’ diameters were calculated by means of image analysis: about 100 beads were sampled, and diameters were obtained from their digital picture. Same went for xerogels, cryogels, and aerogels. Percentage shrinkage (Sh%) was calculated as:

(1) $$\mathrm{Sh}\%=\frac{d_{in}-d_{fin}}{d_{in}}\cdot 100,$$

In Equation (1), d_in_ and d_fin_ stand for initial and final mean diameter of the beads, respectively prior and after manipulation or processing. MATLAB R2024b was used to analyze images and produce particle size distribution of the sampled particles.

Field Emission Scanning Electron Microscopy (FESEM, Carl Zeiss Supra 35, Oberkochen, Germany) observations were carried out once samples were cryofractured in liquid nitrogen and coated with a thin layer of gold (thickness 250 Å) using an Agar Auto Sputter Coater (mod. 108 A, Stansted, UK). Accelerating voltage was set at 30 keV. At least three samples per composition were observed, and analysis was carried out on different points of the beads to ensure morphological homogeneity across the section. Pore size distribution was also computed by means of image analysis, performed using MATLAB R2024b, by means of which image segmentation was performed applying Otsu’s method. Data were described using an inverse Gaussian probability density function, chosen to describe data distributions that display enhanced skewness.

Water uptake measurements were carried out by noting sample initial weight and submerging it in distilled water, until equilibrium or dissolution was reached. Beads have been weighed at regular intervals of time, after being sieved and excess water gently removed with tissue paper.

Fourier Transform Infrared Spectroscopy (FT-IR) was performed using an IR-Tracer 100 (Shimadzu, Kyoto, Japan) system operated in the range 500–4000 cm^−1^, at a resolution of 2 cm^−1^. The KBr pellet method was adopted for this analysis.

#### 4.2.3. Naringin Release Tests

Prior to drug release tests, phosphate buffer solution (PBS) was prepared. Its pH was changed using HCl or NaOH 0.1 M aqueous solutions, to achieve a pH of 7.4 or 6.8 for small or colonic fluids simulations [45]. Naringin concentration was monitored at regular intervals of time by a UV-vis spectrophotometer, whose detection wavelength was set at 272 nm: calibration curves were obtained for both simulated environments. For the simulated environment at pH = 7.4, the slope of the linear relationship between absorbance and concentration was 0.02904 (R^2^ = 0.9986); at pH = 6.8, it was 0.03647 (R^2^ = 0.9999). Cumulative concentration points were thus extracted and normalized with respect to the equilibrium—i.e., steady state—concentration value (C_eq_), equal for all the three kinds of samples. To perform the in-series release experiments, 200 mL of PBS at pH = 7.4 was used first; 50 mg of sample were located in this PBS, and temperature kept constant at 37 ± 0.2 °C for 4 h. After this time was over, fresh PBS at pH = 6.8 was replaced and the operation repeated until a plateau value was reached.

## Figures and Tables

**Table 1:** Shrinkage values for the produced Alg/k-Car blends.

Drying Technique	Alg/k-Car, *v*/*v*	D_fin_, mm	Shrinkage, %
Oven drying	100/0	0.76 ± 0.14	69%
	70/30	0.82 ± 0.16	70%
	50/50	0.83 ± 0.19	70%
	30/70	0.89 ± 0.22	69%
	0/100	N.A.	N.A.
Freeze drying	100/0	1.44 ± 0.28	42%
	70/30	1.51 ± 0.36	44%
	50/50	1.51 ± 0.42	46%
	30/70	1.82 ± 0.40	37%
	0/100	N.A.	N.A.
Supercritical drying	100/0	1.95 ± 0.16	22%
	70/30	2.01 ± 0.20	26%
	50/50	2.00 ± 0.26	28%
	30/70	2.06 ± 0.32	28%
	0/100	1.64 ± 0.36	45%

**Table 2:** Aerogels’ maximum water uptake capacity, and erosion onset time.

Alg/k-Car, *v*/*v*	Max Water Uptake, g/g	Erosion Onset Time, min
100/0	4.30	180
50/50	50.60	120
0/100	63.77	60

## Data Availability

The original contributions presented in this study are included in the article. Further inquiries can be directed to the corresponding author.
